# An ancient role for nitric oxide in regulating the animal pelagobenthic life cycle: evidence from a marine sponge

**DOI:** 10.1038/srep37546

**Published:** 2016-11-22

**Authors:** Nobuo Ueda, Gemma S. Richards, Bernard M. Degnan, Alexandrea Kranz, Maja Adamska, Roger P. Croll, Sandie M. Degnan

**Affiliations:** 1 School of Biological Sciences, University of Queensland, Brisbane QLD 4072, Australia; 2 Department of Physiology & Biophysics, Dalhousie University, Halifax NS B3H 4R2, Canada

## Abstract

In many marine invertebrates, larval metamorphosis is induced by environmental cues that activate sensory receptors and signalling pathways. Nitric oxide (NO) is a gaseous signalling molecule that regulates metamorphosis in diverse bilaterians. In most cases NO inhibits or represses this process, although it functions as an activator in some species. Here we demonstrate that NO positively regulates metamorphosis in the poriferan *Amphimedon queenslandica*. High rates of *A. queenslandica* metamorphosis normally induced by a coralline alga are inhibited by an inhibitor of nitric oxide synthase (NOS) and by a NO scavenger. Consistent with this, an artificial donor of NO induces metamorphosis even in the absence of the alga. Inhibition of the ERK signalling pathway prevents metamorphosis in concert with, or downstream of, NO signalling; a NO donor cannot override the ERK inhibitor. *NOS* gene expression is activated late in embryogenesis and in larvae, and is enriched in specific epithelial and subepithelial cell types, including a putative sensory cell, the globular cell; DAF-FM staining supports these cells being primary sources of NO. Together, these results are consistent with NO playing an activating role in induction of *A. queenslandica* metamorphosis, evidence of its highly conserved regulatory role in metamorphosis throughout the Metazoa.

Most marine invertebrate phyla include species with a biphasic life cycle comprised of a small, planktonic larval form that settles onto the benthos and metamorphoses into a morphologically distinct juvenile^1^,^2^,^3^. Settlement and metamorphosis typically requires that the larva first obtains “competence” to be able to detect and respond to a local inductive environmental cue^4^,^5^. Acquisition of competence may require that the larva first encounters a broad-scale environmental condition indicative of a generally appropriate habitat^6^,^7^. A competent larva in the presence of an appropriate local inductive cue will generally settle and initiate metamorphosis. In eumetazoan marine invertebrate larvae, this is achieved with the aid of neurosensory systems that are finely tuned to detect precise locations most likely to ensure successful postlarval development, juvenile growth and ultimately adult reproduction^5^,^8^. Typically, activation of neurosensory cells by a specific environmental cue results in the release of neuroactive chemicals in the nervous system, which in turn activate a cascade of intercellular signalling events leading initially to behavioural changes at settlement, and subsequently to both morphogenetic and further behavioural changes at metamorphosis^9^,^10^,^11^,^12^,^13^.

Of the signalling systems so far identified as regulators of settlement and metamorphosis, many are not conserved beyond the taxonomic level of family, or sometimes even genus^14^,^15^,^16^,^17^,^18^,^19^. An exception is nitric oxide (NO), a gaseous signalling molecule that has been implicated in repressing settlement and metamorphosis in a wide range of marine bilaterians, including ascidians^20^,^21^, polychaetes^22^, echinoderms^23^, crustaceans^24^ and gastropods^25^,^26^,^27^,^28^. NO activity in settling sea urchin larvae appears to be localised to neurons in the post-oral ciliary band that have a chemosensory function, consistent with the conserved role for NO in animal chemosensory pathways more generally^12^. In all of these bilaterian cases, a reduction in the level of endogenously synthesised NO appears sufficient to either alone initiate metamorphosis, or at least to enhance the response to an inductive exogenous cue. The phylogenetic diversity of taxa in which this has been experimentally observed has led to the proposition that NO maintains larvae in a prepared state for metamorphosis^25^ and that this role for NO may be a highly conserved feature of marine invertebrate settlement and metamorphosis^29^,^30^. However, there are documented cases in ascidians and a gastropod of NO playing a positive role in regulating the initiation and rate of metamorphosis^31^,^32^,^33^,^34^, raising the possibility that differing roles of NO may be associated with different animal life histories and ecologies^30^.

NO is an ancient signalling molecule used not only by animals, but also by bacteria and non-metazoan eukaryotes^35^,^36^,^37^. In animals, NO is primarily synthesised by nitric oxide synthase (NOS)^38^. It mediates a wide range of physiological responses, often via the activation of its primary receptor, soluble guanylyl cyclase (sGC)^39^,^40^,^41^,^42^, although it can also directly activate other signalling pathways by protein S-nitrosylation^43^ and nitration^44^. Importantly, NO appears to have a very deep ancestral role in regulating the timing of life cycle transitions in response to changes in environmental conditions. This role has been documented across the tree of life, in bacteria, slime moulds, fungi, plants and animals^45^,^46^,^47^,^48^,^49^,^50^, which clearly raises the possibility that NO was involved in regulating the major life cycle transition from pelagia to benthos even in the earliest animals. To investigate this, we explore here for the first time the role of NO in metamorphosis in a non-bilaterian animal, namely the haplosclerid demosponge, *Amphimedon queenslandica*^51^ that has a typical pelagobenthic life cycle^3^,^52^.

*A. queenslandica* naturally release larvae on a daily basis^3^,^53^,^54^. These larvae acquire competency by 4–6 hour post emergence (hpe)^3^,^53^,^54^, at which time they readily settle and metamorphose on the articulated coralline algae (CA) *Amphiroa fragilissima*^55^. Although sponges lack neurons^56^,^57^, they have the ability to sense their surroundings and to coordinate behaviours in response to those surroundings^58^,^59^,^60^. The presence of NOS or NO activity has been reported previously in demosponges, although not in relation to settlement and metamorphosis^61^,^62^,^63^. Here, we provide evidence that NO does indeed regulate settlement and the initiation of metamorphosis in *A. queenslandica* by demonstrating the effects of small molecules that (i) inhibit NOS activity, (ii) scavenge NO and (iii) generate exogenous NO.

We find that NO appears to induce, rather than represses, settlement and metamorphosis as has been observed in some ascidians and molluscs^31^,^32^,^33^. As has been documented in the ascidian *Ciona*^33^,^44^,^64^, NO signalling appears to work via – or in concert with – the ERK signalling pathway in *A. queenslandica*. Consistent with this positive regulation of metamorphosis, we find that *NOS* gene expression increases in epithelial, globular and sub-epithelial cells as larvae develop competence to settle and metamorphose. NO is enriched in these cells in larvae and this enrichment continues into the start of metamorphosis.

## Results

### Nitric oxide induces metamorphosis of *Amphimedon queenslandica* larvae

At 25 °C, *Amphimedon queenslandica* larvae become competent to respond to an inductive cue associated with the coralline algae *Amphiroa fragilissima* within 4–6 hours after emerging from their parent^3^. *A. fragilissima* is a potent inducer of settlement and metamorphosis, with over 90% of competent larvae typically settling on its surface just 1–2 hours after first contact^55^. In contrast, larvae maintained in 0.2 μm filtered sea water (FSW) – in the absence of any inductive cues – rarely settle, even when maintained for 24 hours or more^3^,^55^. These contrasting effects on competent *A. queenslandica* larvae are ideal for investigating mechanisms underlying the induction and inhibition of settlement and metamorphosis.

**Figure 1:**
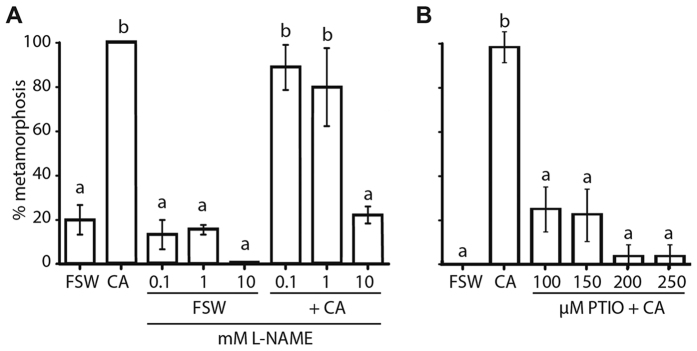
Effect of L-NAME and PTIO on metamorphosis of *Amphimedon queenslandica*. (**A**) Larvae subjected to 0.1, 1 and 10 mM L-NAME at competence (4–6 hpe) and then either reared in FSW or in the presence of *A. fragilissima* (+CA). 10 mM L-NAME significantly inhibits *A. fragilissima*-induced metamorphosis. (**B**) Larvae were subject to 100–250 μM PTIO at competence and then reared in the presence of *A. fragilissima* (+CA). All concentrations of PTIO significantly reduce *A. fragilissima*-induced rates of metamorphosis. Data are presented as the mean percentage of larval metamorphosis ± SEM. Letters above error bars indicate statistically significant differences (P < 0.05), as determined by one-way analysis of variance and Tukey’s HSD *post hoc* testing.

Competent larvae exposed to 10 mM L-nitroarginine methyl ester (L-NAME), which is a competitive inhibitor of NOS, 15 minutes prior to being exposed to *A. fragilissima* were significantly inhibited from initiating and undergoing metamorphosis, even by 24 hours post-induction (hpi) (Fig. 1A). These L-NAME-treated larvae remained in a normal free-swimming state; no mortality was observed. Likewise, the NO scavenger 2-(4-carboxyphenyl)-4,5-dihyrdo-4,4,5,5-tetramethyl-1H-imidazolyl-1-oxy-3-oxide (PTIO) inhibited larvae from initiating metamorphosis in a concentration-dependent manner, with all concentrations over 100 μM significantly reducing the number of individuals metamorphosing in the presence of the inductive coralline algae (Fig. 1B). PTIO-treated larvae also displayed normal swimming behaviour and survival.

**Figure 2:**
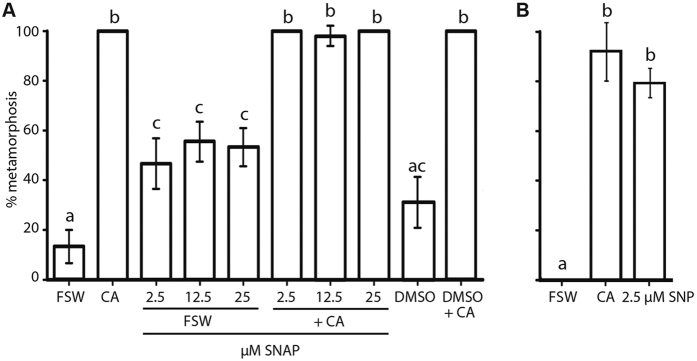
Effect of SNAP and SNP on metamorphosis of *Amphimedon queenslandica*. (**A**) Larvae subjected to 2.5–25 μM SNAP at competence and reared in FSW undergo a significantly higher rate of metamorphosis than the FSW control but not higher than the DMSO control. In the presence of *A. fragilissima* (+CA), rates of SNAP-treated larvae do not differ from +CA controls. (**B**) Larvae exposed to 2.5 μM SNP at competence and reared in FSW have significantly higher rates of metamorphosis than the FSW control, similar to the *A. fragilissima* controls. Data are presented as the mean percentage of larval metamorphosis ± SEM. Letters above error bars indicate statistically significant differences (P < 0.05), as determined by one-way analysis of variance and Tukey’s HSD *post hoc* testing.

Application of the exogenous NO donor sodium nitroprusside (SNP; used at 2.5 μM) induced significantly higher levels of metamorphosis than the FSW control, and in fact induced metamorphosis equivalent to *A. fragilissima* (Fig. 2). In contrast, a different exogenous NO donor, namely S-Nitroso-N-acetylpenicillamine (SNAP, used at 2.5, 12.5 and 25 μM) did not induce metamorphosis above levels in the DMSO control, although its presence had no effect on the ability of *A. fragilissima* to induce high rates of metamorphosis (Fig. 2). In all treatments, larvae and postlarvae behaved and developed normally.

### Nitric oxide signalling activates the MAPK pathway in *A. queenslandica*

**Figure 3:**
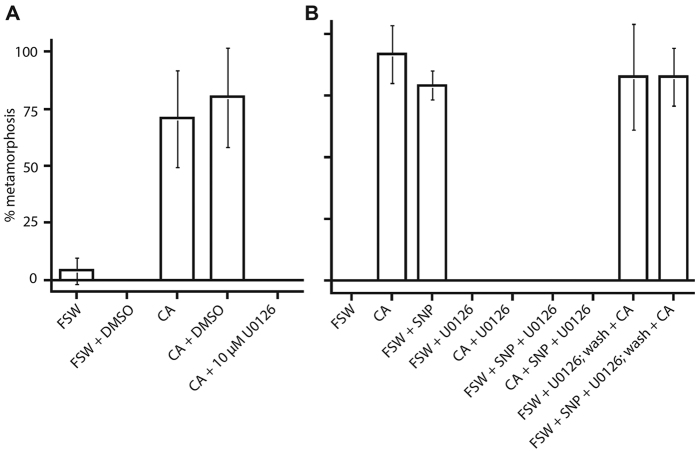
Inhibition of MAPK signalling prevents *A. queenslandica* metamorphosis. (**A**) Larvae subjected to 10 μM U0126 are inhibited from initiating metamorphosis, even in the presence of the inductive coralline alga; an equivalent concentration of 0.1% DMSO does not inhibit metamorphosis. (**B**) Larvae subjected to NO donor, 2.5 μM SNP, undergo metamorphosis at rates similar to coralline alga controls. MAPK inhibitor U0126 inhibits metamorphosis when larvae are in the presence of SNP, coralline algae or both. Larvae removed from U0126, washed in FSW and subjected to the inductive coralline alga undergo high rates of normal metamorphosis.

The MAPK (mitogen-activated protein kinase)/ERK (extracellular signal-regulated kinases) pathway is involved in a wide range of developmental and physiological signalling events and is known both to activate NO production and to be activated by NO, including in ascidian metamorphosis^33^,^44^,^64^. ERK can be selectively inhibited by U0126. In *A. queenslandica*, we found that experimental application of 10 μM U0126 to competent larvae completely inhibits settlement and metamorphosis, even in the presence of *A. fragilissima* (Fig. 3A). This strongly suggests that the MAPK/ERK pathway is necessary for the initiation of metamorphosis. To address whether ERK signalling potentially lies upstream or downstream of NO signalling, we combined U0126 treatment with 25 μM SNP, which is a source of exogenous NO. We found that U0126-treated larvae were unable to settle and metamorphose in the presence of SNP alone, *A. fragilissima* alone, or both together (Fig. 3B). These results are consistent with ERK signalling being necessary for settlement and metamorphosis, and potentially occurring downstream of the metamorphosis-inducing NO signal. Further, when U0126-treated larvae were subsequently washed with FSW and re-exposed to the coralline algae cue, they settled at rates similar to normal untreated larvae indicating the U0126 is not acting as a general toxin (Fig. 3B).

### *A. queenslandica* has a single *NOS* gene that is structurally similar to other metazoan NOS

A single *NOS* gene, hereafter referred to as *AqNOS*, is present in the *A. queenslandica* genome^65^,^66^. Both the protein domain organisation and the intron/exon organisation of *AqNOS* are highly similar to that of other metazoan NOSs, in accordance with the high degree of conservation discussed in ref. 67. Indeed many of exon-intron boundaries are conserved between *A. queenslandica* and bilaterians, although, as is true for this sponge genome in general, intron sizes are comparatively small, with a mean intron size of just 242 bp (Supplementary Fig. 1). AqNOS has a conserved domain architecture that includes an N-terminal PDZ domain as is present in human nNOS, and a split NADPH-ribose binding site as is present in the gastropod mollusc *Lymnaea stagnalis* (Supplementary Fig. 2); specifically, there is a 129 residue insert in the NADPH-ribose binding site compared to the 84 amino acid insert in the *L. stagnalis* gene^68^. The C-terminal end of AqNOS is relatively long compared with other animal NOS proteins (Supplementary Fig. 2).

### *AqNOS* gene expression supports a positive regulatory role of NO in *A. queenslandica* metamorphosis

**Figure 4:**
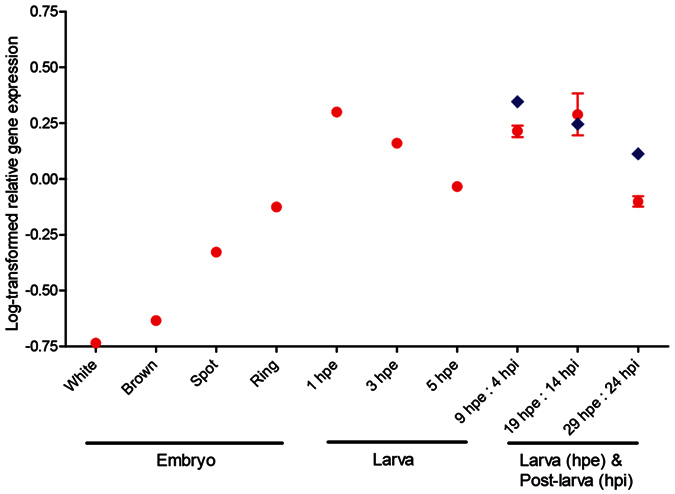
*AqNOS* expression during *Amphimedon queenslandica* development. *AqNOS* transcript abundance measured by qRT-PCR on pools of ~40 embryos still in brood chambers, larvae that are still swimming (red circles) and postlarvae that have settled and initiated metamorphosis (blue diamonds). White stage embryos are undergoing cleavage; brown embryos are commencing cell migration leading to two cell layers; spot stage embryos have two distinct layers and an obvious anteroposterior polarity; 1 and 3 hpe (hours post-emergence) are precompetent swimming larvae; 5+ hpe are competent swimming larvae; and 4, 14 and 124 hpi (hours post-induction) are metamorphosing postlarvae (for further details on developmental stages see ref. 70). Results are presented as log-transformed mean ± SEM of three technical replicates; the SEM was so small for most samples that it is not visible at the scale depicted.

As measured by quantitative RT-PCR, *AqNOS* transcript levels increase continuously through embryogenesis, eventually peaking in free-swimming larvae at 1 hpe (Fig. 4). Transcript abundance then gradually decreases towards competency (5 hpe), but still remains well above embryonic levels. In larvae that are not induced to settle, the transcript abundance increases after competency, reaching a second peak of expression at 19 hpe, and then decreases again by 29 hpe (Fig. 4). In postlarval samples (those induced to settle and metamorphose), peak *AqNOS* expression occurs by 4 hpi, at the very start of metamorphosis, and is followed by a gradual decrease during postlarval development.

**Figure 5:**
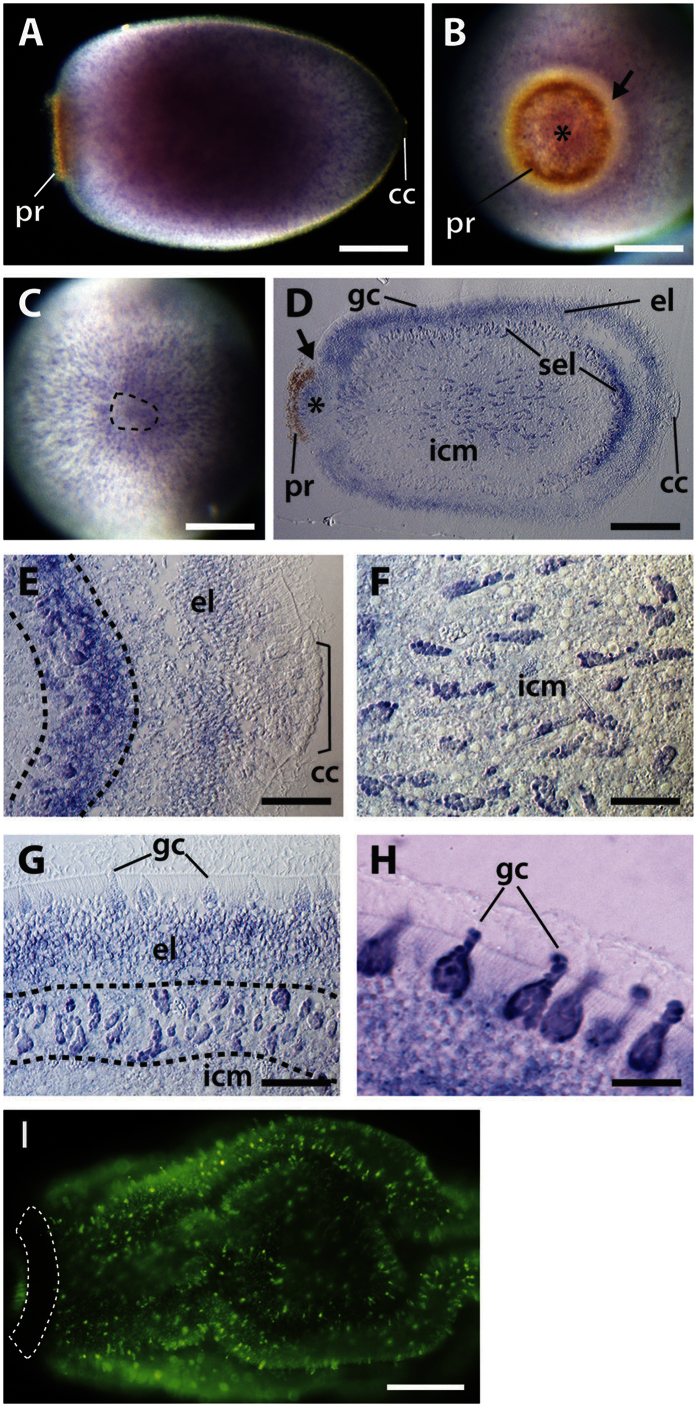
Localised expression of *AqNOS* and endogenous nitric oxide in *Amphimedon queenslandica* larvae. (**A–H**) *AqNOS* gene expression detected by *in situ* hybridisation: (**A–C**) whole mounts, (**C–H**) sections. (**I**) DAF-FM stained larva. Posterior to left except in panels B and C, which show posterior and anterior views, respectively. (**A**) Lateral view showing posterior pigment ring (pr) and anterior cuboidal cells (cc). The ‘salt-and-pepper’ staining indicates localisation of *AqNOS* transcripts to globular cells embedded in outer epithelial layer, and in underlying cell layers. (**B**) Posterior view with staining inside the pigment ring (asterisk) and in a ‘salt-and-pepper’ pattern in the lateral epithelium, but not in the pigment ring itself, or in the adjacent photosensory epithelium (arrow). (**C**) Anterior view showing *AqNOS* transcripts enriched in globular cells but not in anterior cuboidal cells inside the dotted line; there is staining in cells underlying cuboidal cells. (**D**) Longitudinal section through a larva revealing *AqNOS* expression in spherulous cells in inner cell mass (icm) and subepithelial layer (sel), in columnar epithelial and globular cells (gc) in the epithelial layer (el), and in globular cells inside the pigment ring (asterisk), but not in epithelial cells outside the pigment ring (arrow). (**E–H**) Higher magnifications of sections: (**E**) Larval anterior showing *AqNOS* expression primarily in epithelium and spherulous cells in the subepithelial layer, bordered by dashed lines, but not in anterior cuboidal cells; (**F**) Larval inner cell mass showing *AqNOS* transcripts enriched in a subset of spherulous cells but not in other cell types; (**G**) Lateral section showing *AqNOS* expression in columnar epithelial and globular cells in epithelial layer, and spherulous cells in subepithelium, bordered by dashed lines; (**H**) Higher magnification of a more heavily stained larval epithelium showing *AqNOS* transcripts localised to globular cells and to basal portion of columnar epithelium. (**I**) Endogenous nitric oxide, detected using the NO-specific indicator DAF-FM, enriched in globular cells (large, bright green cells dotting the larval surface) and less so in epithelial cells, in a larva just commencing metamorphosis; posterior pigment ring is bordered by dashed lines. Scale bars: (**A**–**D**), I, 100 μm; E-G, 20 μm; F, 10 μm.

Using whole mount *in situ* hybridisation, we detect *AqNOS* transcripts in multiple larval cell types, specifically (i) globular cells that are interspersed on the larval surface between columnar epithelial cells^69^; (ii) the columnar epithelial cells themselves, which form the outer surface layer of the sponge larva; and (iii) spherulous cells that mainly comprise the sub-epithelial layer but also are sparsely distributed in the inner cell mass of the larva (Fig. 5). The expression of *AqNOS* in the epithelium is broad but does not include anterior cuboidal cells, for which a function has yet to be ascribed, or the posterior pigment ring cells and adjacent photosensory cells with long-cilia^70^ (Fig. 5B–E). In the columnar epithelium, *AqNOS* transcripts are enriched in the basal part of the cells; it is unclear if flask cells, which are interspersed amongst the columnar cells in the anterior third of the larva^55^, express *AqNOS*. In the inner cell mass, which is comprised of a number of described and undescribed cell types^71^, we see only two cell types with detectable levels of *AqNOS* transcript; these are spherulous cell types similar in morphology to the globular cells, and subepithelial spherulous cells (Fig. 5D,F).

To confirm that *AqNOS*-expressing cells are the primary source of NO in larvae and at the initiation of metamorphosis, we stained larvae with 4-amino-5-methylamino-2′,7′-difluoro-fluorescein diacetate (DAF-FM), which detects endogenous NO. The resultant DAF-FM staining pattern (Fig. 5I) followed closely the *AqNOS in situ* hybridisation pattern (Fig. 5A–H); signals from cells in the inner cell mass or subepithelial layer could not be resolved with this technique. The DAF-FM signal was brightest in globular cells dotting the surface of the larva (Fig. 5I). As in the *AqNOS in situ* hybridisation, there was no apparent difference in staining intensity of these cells along the anterior-posterior axis (Fig. 5I). NO was also detected in epithelial cells, although DAF-FM staining was markedly less in these cells, again reflecting the *AqNOS in situ* hybridisation pattern (Fig. 5). Tracing DAF-FM staining in larvae over the first hour of metamorphosis, during which time the larva attaches to the substratum and begins to flatten into the juvenile body plan, revealed no changes in the overall pattern of NO; that is, through the first hour of metamorphosis, NO appeared to remain highest in globular cells and detectable in the epithelium (Supplementary Movie 1).

**Figure 6:**
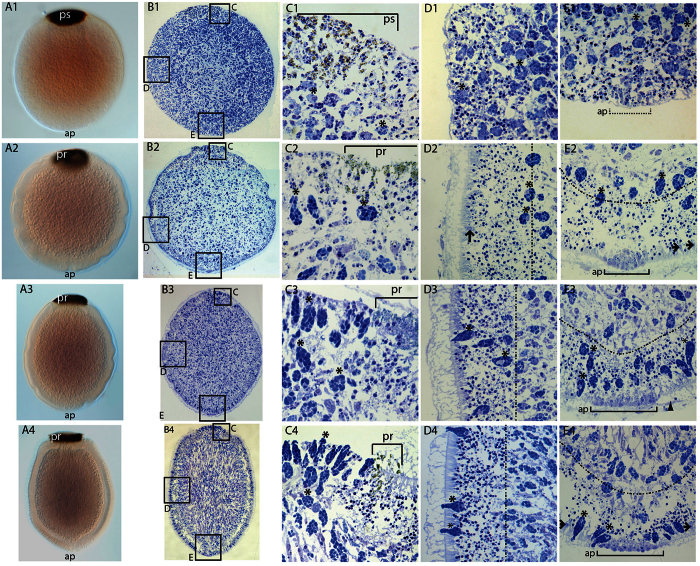
Ontogeny of spherulous and globular cells indicated by toluidine blue staining. (**A1–E1**) Spot stage embryo. (**A2–E2**) Early ring stage embryo. (**A3–E3**) Late ring (pre-hatching) stage embryo. (**A4–E4**) Pre-competent larva. (**A1–A4**) Whole mount embryos and larvae. (**B1–B4**) Sectioned embryos and larvae. Boxes correspond to respective C –E micrographs. (**C1–C4**) Higher magnification of posterior portion of embryos and larvae. (**D1–D4**) Higher magnification of lateral portion of embryos and larvae. (**E1–E4**) Higher magnification of anterior portion of embryos and larvae. ap, anterior pole; pr, pigment ring; ps, pigment spot; dashed line denotes boundary between outer epithelial layer and subepithelial layer; stars denote representative spherulous and globular cells; arrows in D2, E2 and E4 denote flask cells.

*In situ* hybridisation of larvae revealed *AqNOS*-expressing globular cells apparently in the process of migrating out of the sub-epithelial layer – where they are classified as spherulous cells – to the outer epithelial layer, a migration that has been observed previously for globular cells during larval development^69^,^70^,^71^,^72^. The detection of *AqNOS* and NO in these cells, at a time coincident with larval responsiveness to NO, implies that globular cells are a source of endogenous NO, and involved in the process of larval settlement and metamorphosis. In their final location around the periphery of the larva, globular cells are tightly packed with large electron-dense granules, basal nuclei and apices that protrude outwards above the columnar epithelium^73^. Based on this distinctive morphology, we were able to trace the ontogeny of globular cells using toluidine blue (Fig. 6). At the spot stage of development (Supplementary Fig. 3), a population of spherulous cells that display similar staining characteristics to the larval globular cells are throughout the embryo (Fig. 6D1,E1). By the ring stage (Supplementary Fig. 3), the most outer layer of the embryo becomes clear of the spherulous cell population (Fig. 6A2–E2), which is now more densely visible at the interface between inner and outer layers, effectively forming a third layer in the embryo (Fig. 6D2). As the ring continues to form and the late embryo starts to elongate into the larval form (Fig. 6A3–E3), the spherulous cells migrate from the sub-epithelial layer and either intercalate into the ciliated columnar epithelium or localise at the posterior pole. Once they are in their final location, we now consider these cells to be globular cells. At hatching, globular cells are evident around the entire periphery of the *A. queenslandica* larva (Fig. 6A4–E4), except at the very anterior pole (Fig. 6E4) and amongst the pigmented cells at the posterior pole (Fig. 6C4). In the swimming larva, spherulous cells continue to migrate to the surface, and thus continue to increase the total number of externally-localised globular cells. By the time the larva attains competence, globular cells appears to be at higher density in the anterior larval epithelium (Fig. 6A4–B4).

## Discussion

Inhibiting NOS activity by the application of 10 mM L-NAME, or by the scavenging of released NO with PTIO, prevents *Amphimedon queenslandica* larvae from initiating metamorphosis even in the presence of the highly inductive coralline algae *Amphiroa fragilissima*. Consistent with this, the artificial elevation of NO levels in FSW, via the application of SNP, induces high rates of *A. queenslandica* larval settlement and metamorphosis, even in the absence of *A. fragilissima*. Together, these results indicate that NO signalling is necessary – and perhaps also sufficient – for the induction of settlement and initiation of metamorphosis of *A. queenslandica* larvae. The activating role of NO in *A. queenslandica* settlement and metamorphosis contrasts with observations in a range of bilaterian larvae where NO appears to play a repressive or inhibitory role^20^,^21^,^22^,^23^,^24^,^25^,^26^,^27^,^28^, but is similar to other species where it appears to play an inductive or stimulatory role^30^,^31^,^32^,^33^,^34^.

*A. queenslandica* has a single NOS gene (*AqNOS*) that encodes a protein domain architecture that is deeply conserved with the rest of the animal kingdom, yet includes a number of uncommon metazoan features, including a N-teminal PDZ domain and a split NADPH-ribose binding domain. *AqNOS* transcript abundance increases during embryogenesis to reach maximum levels in larvae, in which expression is spatially enriched in the ciliated columnar epithelium, and in globular and spherulous cells. These temporal and spatial expression profiles support a role for NOS in regulating settlement and metamorphosis in *A. queenslandica*. Corroborating these gene expression analyses is the detection, using the DAF-FM indicator, of comparable relative levels of NO in columnar epithelial and globular cells. Globular cells, which appear in *A. queenslandica* to be derived from spherulous cells, intercalate into the larval columnar epithelium and have apical protrusions that extend beyond the larval surface. These cells, along with the larval flask cells^55^, have been implicated as having a sensory role, based on their expression of postsynaptic genes, neurogenic factors and signals, and genes in the Toll pathway^74^.

**Figure 7:**
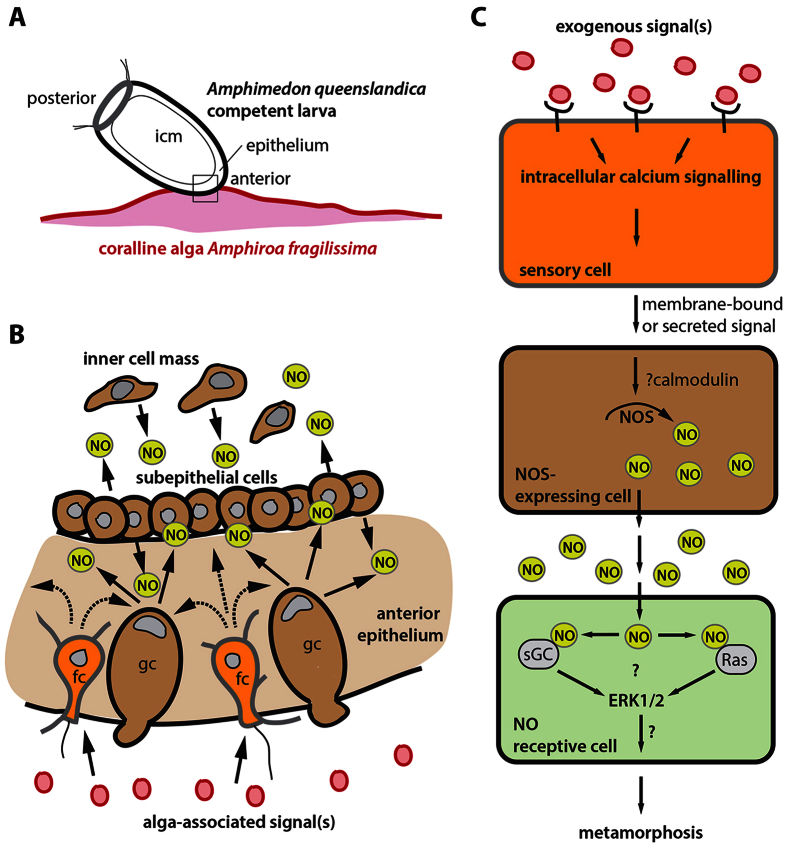
A model for the role of nitric oxide in the induction of larval settlement and metamorphosis. (**A**,**B**) A schematic summarising the potential role of nitric oxide in larval settlement of *Amphimedon queenslandica*. (**A**) The competent *A. queenslandica* larva settles onto a coralline alga, anterior side touching the substratum. (**B**) A close-up of the boxed anterior region shown in (**A**). Exogenous signals associated with the algal surface activate a calcium signalling in flask cells (fc)^55^, which in turn signals to the surrounding cellular environment via membrane bound (e.g. Notch-Delta signalling^71^ or locally diffusible signals (dashed arrows). In *A. queenslandica*, this includes columnar epithelial and globular cells (gc), which express high levels of *AqNOS* (brown). These cells are induced to release NO (arrows) at a rate to induce morphogenetic and physiological changes in other larval cells, including other NOS-expressing cells (brown), leading to larval settlement and the initiation of metamorphosis. MAPK signalling is also required for *A. queenslandica* settlement and metamorphosis (not shown). (**C**) A general model for nitric oxide signalling in induction of larval settlement and metamorphosis. Receptor-activated calcium signalling occurs in sensory cells upon contact with an inductive exogenous cue, resulting in the release of a signalling ligand. This is detected by NOS-expressing cells, possibly by calcium-calmodulin binding to NOS, activating the synthesis of NO. The NO signal can induce cellular changes, including by binding to its receptor soluble guanylyl cyclase (sGC) and S-nitrosylation of members of the Ras-GTPase super family; these may result in the activation of the MAPK/ERK signalling pathway. In bilaterian larvae where NO inhibits or represses the settlement and metamorphosis^20^,^21^,^22^,^23^,^24^,^25^,^26^,^27^,^28^, a different model of NO and NOS regulation is required. icm, inner cell mass.

The common morphology of *AqNOS*-expressing spherulous cells in the inner cell mass and subepithelial layer, and the globular cells on the larval surface, suggests that these cells are ontogenetically related and have a similar role in NO signalling. However, prior direct evidence of a role for globular or spherulous cells in controlling metamorphosis has not existed, and recently another larval cell type – the flask cell – has been implicated in contributing to the regulation of this process^55^. Regardless, the requirement of NO signalling implicates these *NOS*-expressing cells in the regulation of settlement and metamorphosis. Interestingly, globular cells accumulate differentially in the epithelial layer of the anterior half of larvae as they develop competence to settle and metamorphose. This anterior region is also where the flask cells are primarily located, and in fact the anterior third of the larva appears to play a critical role in the induction of metamorphosis^55^. Inhibition of intracellular calcium signalling in flask cells also inhibits metamorphosis^55^, further supporting a functional link between these cells and *NOS*-expressing cells. Together, these results support a model where the anterior part of *A. queenslandica* larvae contacts an environmental cue, which induces an interplay of intra- and intercellular signals in flask and globular cells that results in the release of NO and the initiation of metamorphosis (Fig. 7).

NO is a short-lived, highly diffusible molecular messenger^75^. In poriferans, which lack a nervous system, NO may allow for a coordinated and relatively rapid response to an external signal, as required for the initiation of metamorphosis. Settlement and metamorphosis in *A. queenslandica* is accompanied by a diversity of cellular changes, including widespread apoptosis and transdifferentiation^76^. The highly organised and patterned larva transforms within hours into a less structured “mat” of cells that adhere both to each other and to the substrate^76^. There is a rapid decrease in the number of cell types at this transition, with some larval cell types transdifferentiating into a common pluripotent stem cell type, the archeocyte^53^,^76^. There is no evidence of metamorphosis commencing in a particular part of the larva or via a morphogenetic wave, although the anterior of the larva usually makes first contact with the inductive substratum. Thus, the widespread morphogenetic transformation of the larva that rapidly follows settlement is consistent with the broad expression pattern of *AqNOS*, with the presence of NO in epithelial and globular cells along the entire anteroposterior axis, and with the rapid diffusion of a gaseous molecule such as NO (Fig. 7).

Inhibition of metamorphosis by treatment with U0126, a MAPK inhibitor, is consistent with the ERK pathway also being necessary for the induction of settlement and metamorphosis in *A. queenslandica*. This inhibition is maintained in the presence of the NO donor SNP or the inductive coralline algae *A. fragilissima*, or both, suggesting that the ERK pathway is downstream of the NO signalling event that initiates settlement and metamorphosis. However, we cannot exclude the possibility that this activation of MAPK/ERK occurs separately from the NO signalling pathway. NO signalling is often mediated by the binding of NO to the heme moiety of soluble guanylyl cyclase (sGC), thus activating this enzyme and resulting in an increase in the synthesis of cyclic guanosine 5′ monophosphate (cGMP)^75^. Cyclic GMP in turn (i) activates cGMP-dependent protein kinases (PKG), (ii) activates or inhibits cAMP-specific phosphodiesterases (PDEs), and (iii) opens cyclic nucleotide-gated cation channels, leading to a wide range of context-specific physiological and developmental outcomes in animals, some of which include ERK signalling^29^,^41^,^77^,^78^,^79^. In addition, NO can impact on cell state directly by S-nitrosylation of cysteine residues of signalling pathway components and transcription factors^43^,^80^,^81^, including members of the Ras-GTPase super family^82^,^83^. Thus it is possible that ERK activation may occur via the S-nitrosylation of Ras, or an alternative mechanism, in *A. queenslandica* (Fig. 7). For example, in *Ciona*, an elevation of NO under oxidative-stress-like conditions results in protein nitration, including tyrosine residues in ERK^44^. In this ascidian, NO also down-regulates MAP kinase phosphatases, which results in a increase in ERK signalling, and thus indicates yet another possible means by which NO can increase the signalling activity of this pathway.

### Conclusions and hypothesis

We have provided multiple lines of evidence that support NO being both necessary and sufficient to activate larval settlement and metamorphosis in a marine sponge, *A. queenslandica*. Known NOS inhibitors and NO scavengers inhibit metamorphosis, and an NO donor induces metamorphosis. The localised expression of *AqNOS* and concomitant accumulation of detectable NO together suggest that larval globular cells and columnar epithelial cells are likely sources of NO in the larvae of this sponge (Figs 5 and 7). These cells may be interacting with sensory flask cells^55^ in the larval anterior to control settlement and metamorphosis (Fig. 7). NO typically represses the initiation of metamorphosis in multiple bilaterian marine invertebrates^20^,^21^,^22^,^23^,^24^,^25^,^26^,^27^,^28^,^29^, although there are other reported cases where NO acts as an activator of metamorphosis^30^,^31^,^32^,^33^,^34^. The results presented here for the demosponge *A. queenslandica* phylogenetically extend the role of NO in animal metamorphosis beyond the bilaterians, thus raising the possibility that its action harks back to the last common animal ancestor, and that it has been able to switch between being an activator and repressor of life cycle transitions over the course of animal evolution.

Using the results presented herein to build on previously published studies, we hypothesise more generally that settlement and metamorphosis in marine invertebrates requires first the binding of an environmentally-derived ligand to cell-surface sensory receptors (potentially G-protein coupled receptors - GPCRs) that in turn initiates intracellular calcium signalling (Fig. 7). Since NO is a critical modulator of chemosensory stimuli, especially in olfactory circuits^84^, it has long been proposed that the regulation of NOS can be linked to GPCRs activated by external chemical ligand binding^85^, although it is noteworthy that studies to date have failed to find support for a role of GPCRs in one annelid^86^ and two cnidarian^87^ species.

A current model of GPCR-NOS signalling is that a ligand-bound GPCR complex activates the signalling cascade of phospholipase C-inositol-1,4,5-triphosphate, resulting in the release of Ca^2+^ from the endoplasmic reticulum into the cytosol^85^. In *A. queenslandica*, this may be occurring in globular cells by induction by an exogenous ligand or from a signal originating in flask cells. In support of the latter, there is evidence of calcium signalling in globular cells after an initial release of intracellular calcium in flask cells^55^. Both flask cells and globular cells express Notch and a unique suite of Delta ligands^71^. In addition, flask cells extend filopodial processes above and within the epithelium^55^, raising the possibility that these cell types can interact via this signalling pathway. Regardless of whether direct or indirect, calcium signalling likely allows the activation of NOS, and thus NO production, via the binding of calmodulin^42^,^88^.

As a gaseous molecule, NO can freely and rapidly diffuse across cells and the cellular membrane, and can quickly modify its target molecules by covalent binding^89^. Evidence to date suggests that this target could be either Ras or sGC, both of which can result in activation of the ERK pathway and, ultimately, regulation of the comprehensive morphogenetic changes that constitute metamorphosis (Fig. 7); recent analyses in the ascidian *Ciona* indicate that NO may affect ERK signalling also by direct nitration or inhibition of MAP kinase phosphatases^33^,^44^. Indeed, application of an sGC inhibitor, ODQ (1H-[1,2,4]oxadiazolo[4,3-a]quinozalin-l-one), significantly increases the induction of metamorphosis in several marine invertebrates^20^,^23^,^24^,^25^,^26^,^27^. We suggest that the model outlined in Fig. 7 could provide a framework for future experiments, exploiting available pharmaceutical reagents to manipulate each step, and thus to test the role of NO and its upstream and downstream components in the metamorphosis of a wide range of marine invertebrate species.

## Methods

### *Amphimedon queenslandica* larval cultivation

Adult specimens of the sponge *Amphimedon queenslandica* were collected from Heron Island Reef, Great Barrier Reef, Australia (23° 26′S, 151° 55′E). All embryological material was procured from dissected brood chambers as described in Leys *et al*.^90^. For larval collection, adult specimens were maintained in tanks with gentle flow of ambient seawater. Larvae released naturally during the day^91^ were collected in containers with 100 μm nitex mesh bottoms placed at the outflow. Larvae were collected every hour, allowing the determination of larval ages within 1 hour post emergence (hpe); that is, a 1 hpe sample contains larvae that had emerged between 0 (larvae collected immediately after emergence from its maternal adult) and 1 h (larvae that emerged from the maternal adult shortly after the previous collection). The collected larvae were maintained in 500 mL ambient seawater at 25 ± 0.5 °C for at least 4–5 h to allow them to reach competency^3^ (Supplementary Fig. 3).

### Assessment of NO-manipulating small molecules on metamorphosis

The following small molecules that influence NO levels were used in this study: NOS inhibitor L-nitroarginine-methyl-ester (L-NAME; Sigma), NO scavenger 2-(4-carboxyphenyl)-4,5-dihyrdo-4,4,5,5-tetramethyl-1H-imidazolyl-1-oxy-3-oxide (PTIO; Sigma), and NO donors S-nitroso-N-acetyl-penicillamine (SNAP; Sapphire Bioscience) and sodium nitroprusside (SNP; Sigma). All these chemicals have demonstrated significant effects on marine invertebrate metamorphosis^20^,^21^,^22^,^23^,^24^,^25^,^26^,^27^.

Stock solutions of 0.5 M L-NAME, 2.5 mM PTIO and 2.5 mM SNP were prepared in 0.2 μm filtered sea water (FSW), stored at 4 °C and diluted just prior to the experiment; SNP was prepared just prior to its use. The stock solution of 50 mM SNAP was prepared in dimethyl sulfoxide (DMSO) diluted immediately prior to the experiment. To maintain the steady concentration of NO delivered by SNAP, the solution was renewed every 6 h^25^.

All settlement assays were performed in 6-well 35-mm diameter sterile polycarbonate tissue culture dishes, with 10 ml of FSW per well, and were initiated at 5 hpe, by which time all larvae would be competent (Supplementary Fig. 3). Three replicates of 15 larvae were incubated continuously in wells containing either (1) 10 ml FSW only, (2) 10 ml FSW with freshly prepared shards of coralline algae *A. fragilissima*, which were collected from the Heron Island reef flat and cleaned just prior to each experiment, covering approximately 25% of the bottom of the well or (3) 10 ml FSW containing a one of the above mentioned small molecules either with or without coralline algal shards. Inhibitory molecules L-NAME and PTIO were added to the wells with larvae 15 minutes prior to the addition of *A. fragilissima*. The number of larvae initiating metamorphosis was scored after incubating the culture dishes for 24 h post induction with *A. fragilissima* (24 hpi) in the dark at 25 °C. Postlarvae were deemed to have initiated metamorphosis only if they met all three of the following criteria: (i) were attached to a substrate; (ii) had begun flattening along the anteroposterior axis; and (iii) had begun resorbing their pigment ring^53^. Because of natural biological variation in larval competence and the *A. fragilissima* inducer, all controls were run in every experiment to allow treatments to be related to controls in every cohort.

Settlement and metamorphosis data were analysed by one-way analysis of variance (ANOVA) by testing treatment as a factor. Significant differences among treatments were detected by Tukey’s HSD *post hoc* testing. Prior to the analyses, all data were arcsine-transformed to improve the normal distribution of samples. Levene’s test was performed to assure the homogeneity of variance among treatments. All statistical analyses were performed in R (R Foundation for Statistical Computing). An alpha value of 0.05 was used to determine a significant difference^92^.

### U0126 treatments

A 10 mM solution of the MAPK inhibitor U0126 (Promega, V1121) was prepared in DMSO and further diluted in (FSW) to a final concentration of 10 μM. Competent larvae (5 hpe; Supplementary Fig. 3) were transferred into FSW containing either 10 μM U0126 or 0.1% DMSO controls as described above for NO small molecule treatments. Treated and control larvae were subject to coralline algae or SNP or both 15 minutes later, as described above. To ensure the inhibitory effect of U0126 was not related to toxicity, U0126-treated larvae were washed twice in FSW after a 15 min treatment, and then were subject to coralline algae as described above.

### Analysis of the *A. queenslandica NOS* gene

The nucleotide (XM_011411881) and derived amino acid (XP_011410183) sequences of *A. queenslandica* NOS are available in the NCBI database, where the gene is annotated as an inducible NOS, and at http://metazoa.ensembl.org/Amphimedon_queenslandica/Info/Index where the gene is annotated as Aqu1.226552 based on genome version 1^65^. Using these databases, as well as our most recent in-house automated gene model prediction (Aqu2.1 gene models; see ref. 66), we manually curated a NOS gene model, evaluated intron**–**exon boundaries and domain organization as previously described for other genes^93^, and compared these to the metazoan-wide survey of NOS gene structure by Andreakis *et al*.^67^. Herein we refer to this gene as *AqNOS*.

### Quantitative RT-PCR analysis of *AqNOS* expression

RNA was purified from embryonic, larval, and postlarval stages as previously described^93^,^94^; each developmental stage was represented by a single RNA sample purified from a pool of approximately 40 individual embryos, larvae or postlarvae that were sourced from a pool of 10 different adult sponges. Embryonic staging followed established morphological criteria: white (early blastula), brown (late blastula), spot (concentrated pigment cells are visible at the posterior end), and ring (appearance of conspicuous pigmented ring formation at the posterior end) (Supplementary Fig. 3)^53^,^70^. Larval age was determined by duration (in hours) post emergence from the maternal brood chamber (hpe) as described above. To ensure non-overlap of larval age between samples, only 1, 3, and 5 hpe samples were assayed (Supplementary Fig. 3). To collect postlarval samples, competent larvae (5 hpe) were exposed to live *A. fragilissima* shards in 12 cm petri dishes to induce metamorphosis, and settled postlarvae that had initiated metamorphosis were then sampled at 4, 14 and 24 hpi. Non-induced, still-swimming larval samples of the same developmental age were also collected at 9, 19 and 29 hpe for comparison (Supplementary Fig. 3).

*AqNOS* transcript abundance during normal development was determined by qRT-PCR using the following oligonucleotide primer set: forward 5′-TACGAGGAGCTCACCTACGG-3′ and reverse 5′-TCCTTGGTTTGTGGCATAGC-3′. The geometric mean of two reference genes –*succinate dehydrogenase complex, subunit A* (*SDHA*) (forward 5′-CGGGGAGTGGTAGCTATGAA-3′ and reverse 5′-TGAAACTGTACAAACTCCATGTCT-3′) and *tyrosine 3-monooxygenase/tryptophan 5-monooxygenase activation protein, zeta polypeptide* (*YWHAZ*) (forward 5′- AGCGGAGGGCAAAAAGAA-3′ and reverse 5′-CCTCGGCAAGATAGCGATAGTAGT-3′) was used to normalise the level of transcription for obtaining relative gene expression values. Their stability within and between embryonic, larval, and postlarval stages was confirmed using Genorm software^95^.

The following qRT-PCR reaction parameters were used: initial denaturation 95 °C for 10 min (ramp rate 4.4 °C/sec), and 40–50 cycles of 95 °C for 5 sec (ramp rate 4.4 °C/sec), 58 °C for 10 sec (ramp rate 2.2 °C/sec), and 72 °C for 20 sec (ramp rate 4.4 °C/sec). Melt curve data acquisition was from 55–95 °C with continuous measurement (acquisition/°C = 5) to confirm the purity of PCR product by the presence of a single peak in the temperature melt curve. All samples were run in triplicate to calculate the average values to take technical variations into account. For each primer pair, a standard curve was generated to calculate the efficiency of qRT-PCR using a dilution series from the calibrator sample, which was a mixture of 4 μl (normal development and small molecule exposure) of all undiluted cDNA samples of each experimental set. In addition to the developmental stage cDNAs, a no-template (H_2_O) control and the calibrator sample were included for each qRT-PCR run and for each primer pair. The efficiencies of each primer pair and the cycle threshold of each sample were calculated by the second derivative method using Roche Light Cycler 480 software program. Relative expression ratios were calculated as the ratio of gene expression between the gene of interest and the geometric mean of the two reference genes (normalisation factor) relative to their calibrator sample. The expression ratios and standard errors were calculated using REST-RG beta software version 3^96^.

### Whole mount *in situ* hybridisation analysis

*AqNOS* gene expression patterns were assessed in precompetent (3 hpe) and competent (5 hpe) *A. queenslandica* larvae (Supplementary Fig. 3). Fixation and storage of specimens, and whole mount *in situ* hybridisation (WMISH) were performed as described in Larroux *et al*.^93^,^94^. 798 and 1649 bp fragments of the *AqNOS* cDNA were used as probes (Supplementary Fig. 4). WMISH images were captured with Olympus BX60 using a Nikon Digital Sight DS-U1 camera.

### Detection of endogenous nitric oxide

NO was detected *in vivo* using 4-amino-5-methylamino-2′,7′-difluoro- fluorescein diacetate (DAF-FM) (Molecular Probes) as previously described for the ascidian *Ciona*^21^. Competent larvae (6–8 hpe) were incubated in the dark with 5 μM DAF-FM in FSW for 30 min, and then washed twice and maintained in sea water. Larvae were subsequently viewed and photographed in the process of settling and initiating metamorphosis (Supplementary Movie 1; Fig. 5I)

### Tracing the ontogeny of spherulous and globular cells by staining with toluidine blue

WMISH analysis highlighted the particular relevance to this study of two cell types, namely spherulous and globular cells, that are know from previous work to share an ontogenetic relationship^69^,^70^,^71^,^72^. The distinctive morphology of globular cells^70^,^73^ facilitates the tracing of their ontogeny using toluidine blue. To do so, embryos from spot stage through to pre-competent larvae were fixed in 4% paraformaldehyde + 0.05% glutaraldehyde/1XMOPS buffer, dehydrated, then embedded in Epon epoxy resin and cut into 1 um sections. Sections were stained with a standard toluidine blue solution (0.5% toluidine blue, 1% borax).

## Additional Information

**How to cite this article**: Ueda, N. *et al*. An ancient role for nitric oxide in regulating the animal pelagobenthic life cycle: evidence from a marine sponge. *Sci. Rep.* **6**, 37546; doi: 10.1038/srep37546 (2016).

**Publisher’s note:** Springer Nature remains neutral with regard to jurisdictional claims in published maps and institutional affiliations.

## Supplementary Material

Supplementary Information

Supplementary Movie 1
