# Supplementary material for: An ancient role for nitric oxide in regulating the animal pelagobenthic life cycle: evidence from a marine sponge
Source: Sci Rep. 2016 Nov 22;6:37546. doi: 10.1038/srep37546 (PMC5118744; doi:10.1038/srep37546)
Supplement: Supplementary Information [file srep37546-s1.pdf]

Supplementary information to accompany:

An ancient role for nitric oxide in regulating the animal pelagobenthic life cycle: evidence from a marine sponge

Nobuo Ueda[1,3], Gemma Richards[1], Bernard M. Degnan[1], Alexandrea Kranz[1], Maja Adamska[1,4], Roger Croll[2], and Sandie M. Degnan[1*]

[1] School of Biological Sciences, University of Queensland, Brisbane QLD 4072, Australia
[2] Department of Physiology & Biophysics, Dalhousie University, Halifax NS B3H 4R2, Canada

[*]Corresponding author: s.degnan@uq.edu.au

Current addresses:
[3] Max Planck Institute for Developmental Biology, Tuebingen, Germany
[4] Research School of Biology, Australian National University, Canberra ACT, Australia

This Supplementary Information contains:

Supplementary Fig. 1. Organisation of *AqNOS* and other metazoan *NO* genes. The organisation of *Amphimedon AqNOS*, human (Hs) *iNOS*, *eNOS* and *nNOS*, and the *Drosophila* (Dm) *NOS* genes are shown. Exons are drawn to scale as boxes. These are separated by introns, which are not drawn to scale; sizes are indicated. Exons encoding conserved protein domains and comprising untranslated regions (UTRs) are coloured as per the legend. Conserved exon---intron boundaries are highlighted by a red arrowhead.

Supplementary Fig. 2. Domain architecture of AqNOS and other metazoan NOS proteins. The domain structure of AqNOS, human (Hs) iNOS, eNOS and nNOS, and the *Drosophila* (Dm) and *Lymnaea stagnalis* (Ls) NOS proteins. Protein domains are coloured as per the legend.

Supplementary Fig. 3. Summary of experimental design, developmental timeline and sampling regime.

Supplementary Fig. 4. Nucleotide sequences used to design RNA probes for whole mount *in situ* hybridisation. Grey highlights indicate both the 798 bp probe and the 1649 bp probe, corresponding respectively to position 656 – 1453 and 3082 – 4730 in the open reading frame.

Supplementary Movie 1. Endogenous nitric oxide in a larva initiating metamorphosis. This 22 second time lapse video captures NO localisation over the first hour of a larva when it settles and initiates metamorphosis. The larva was preincubated in DAF-FM to visualise the location of endogenous NO (green). The anterior of the larva is attached to the substratum at the bottom (~7 o'clock) and the posterior of the larva is pointing toward 1 o'clock; the resorbing posterior pigment ring is visible as a black area. NO is primarily observed in globular cells (bright green punctate pattern) and surrounding epithelium.

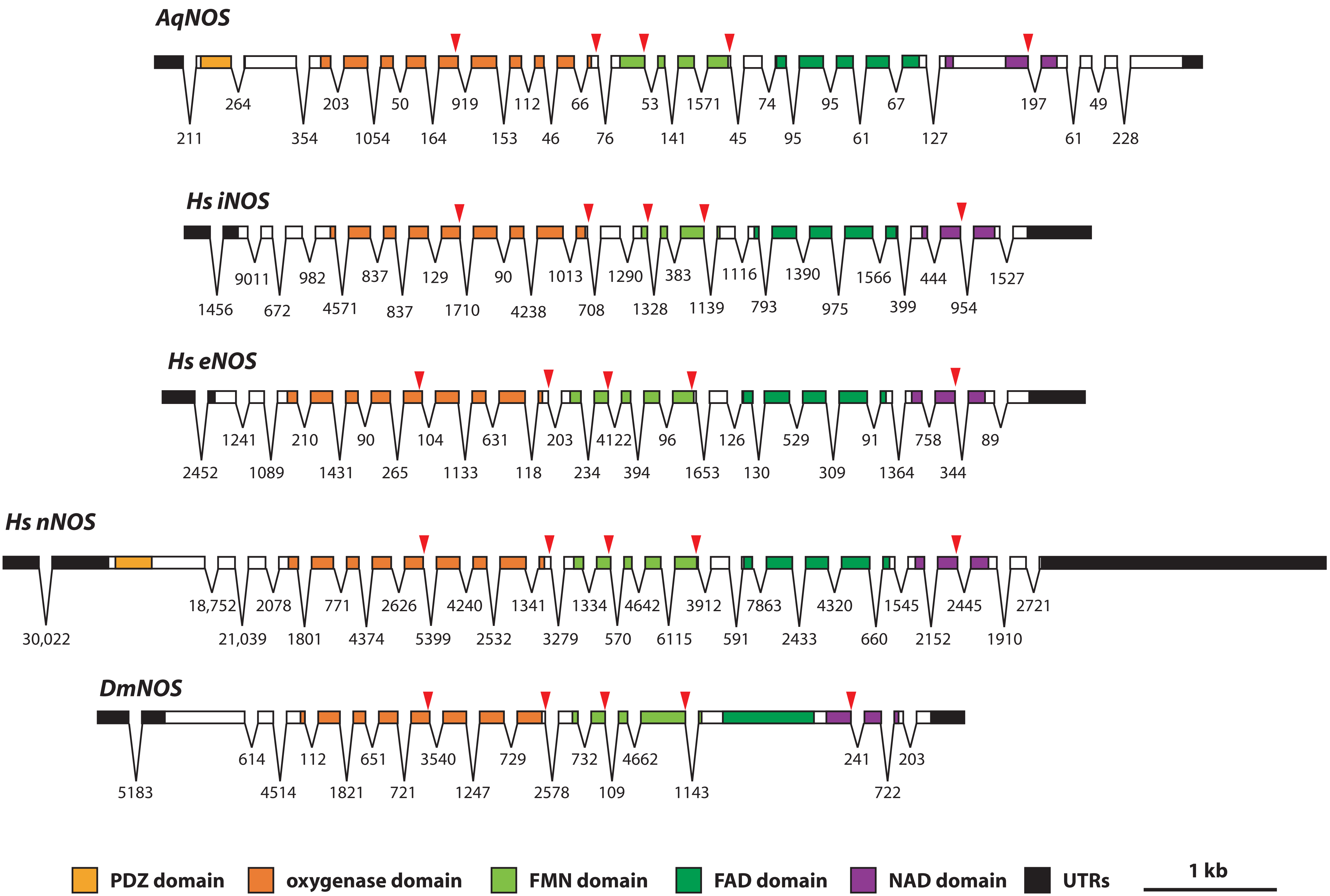
AqNOS
264
203
50
919
112
66
53
1571
74
95
67
197
49
211
354
1054
164
153
46
76
141
45
95
61
127
61
228
Hs iNOS
9011
982
837
129
90
1013
1290
383
1116
1390
1566
444
1527
1456
672
4571
837
1710
4238
708
1328
1139
793
975
399
954
Hs eNOS
1241
210
90
104
631
203
4122
96
126
529
91
758
89
2452
1089
1431
265
1133
118
234
394
1653
130
309
1364
344
Hs nNOS
18,752
2078
771
2626
4240
1341
1334
4642
3912
7863
4320
1545
2445
2721
30,022
21,039
1801
4374
5399
2532
3279
570
6115
591
2433
660
2152
1910
DmNOS
614
112
651
3540
729
732
4662
241
203
5183
4514
1821
721
1247
2578
109
1143
722
PDZ domain
oxygenase domain
FMN domain
FAD domain
NAD domain
UTRs
1 kb

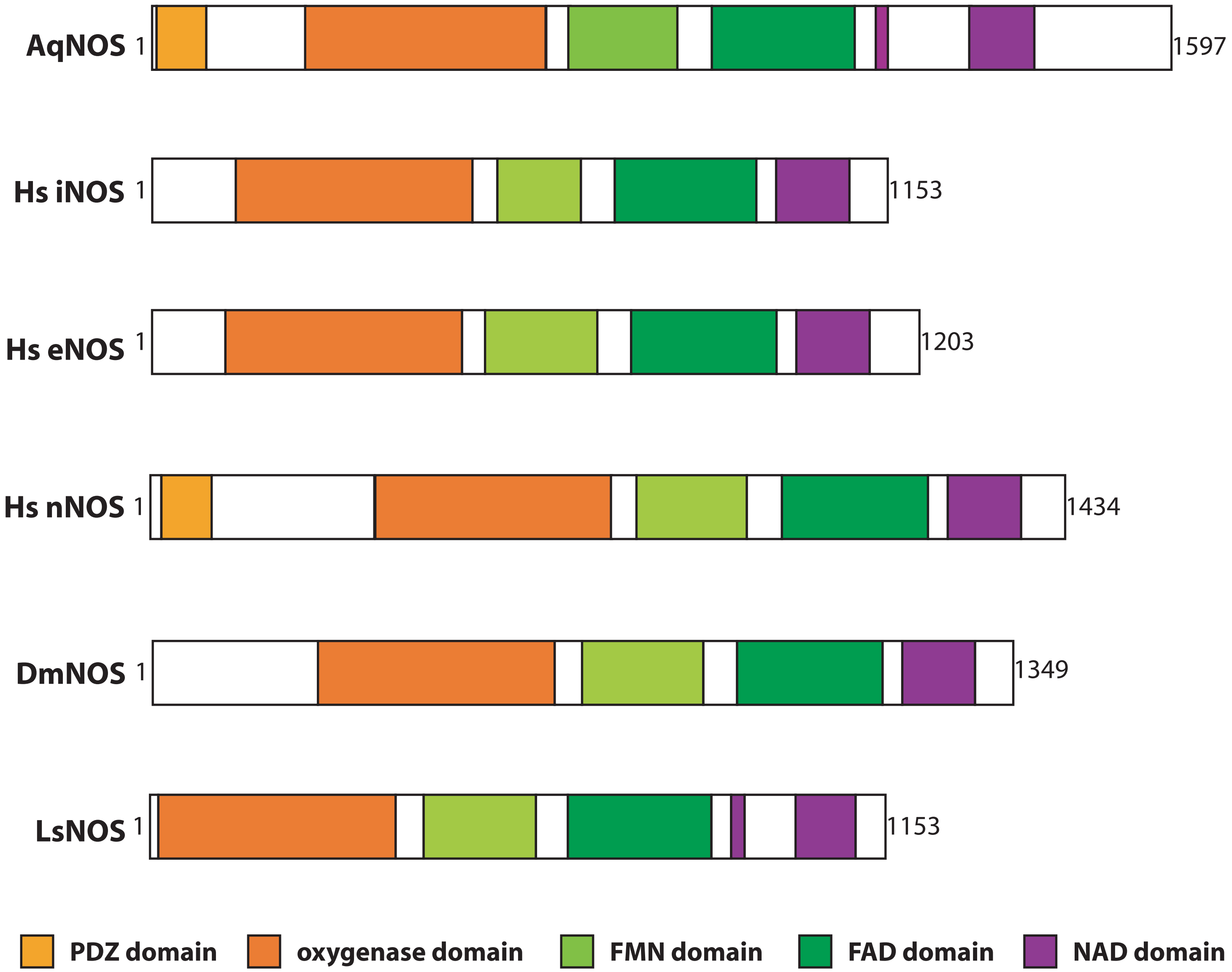
AqNOS 1
1597
Hs iNOS 1
1153
Hs eNOS 1
1203
Hs nNOS 1
1434
DmNOS 1
1349
LsNOS 1
1153
PDZ domain
oxygenase domain
FMN domain
FAD domain
NAD domain

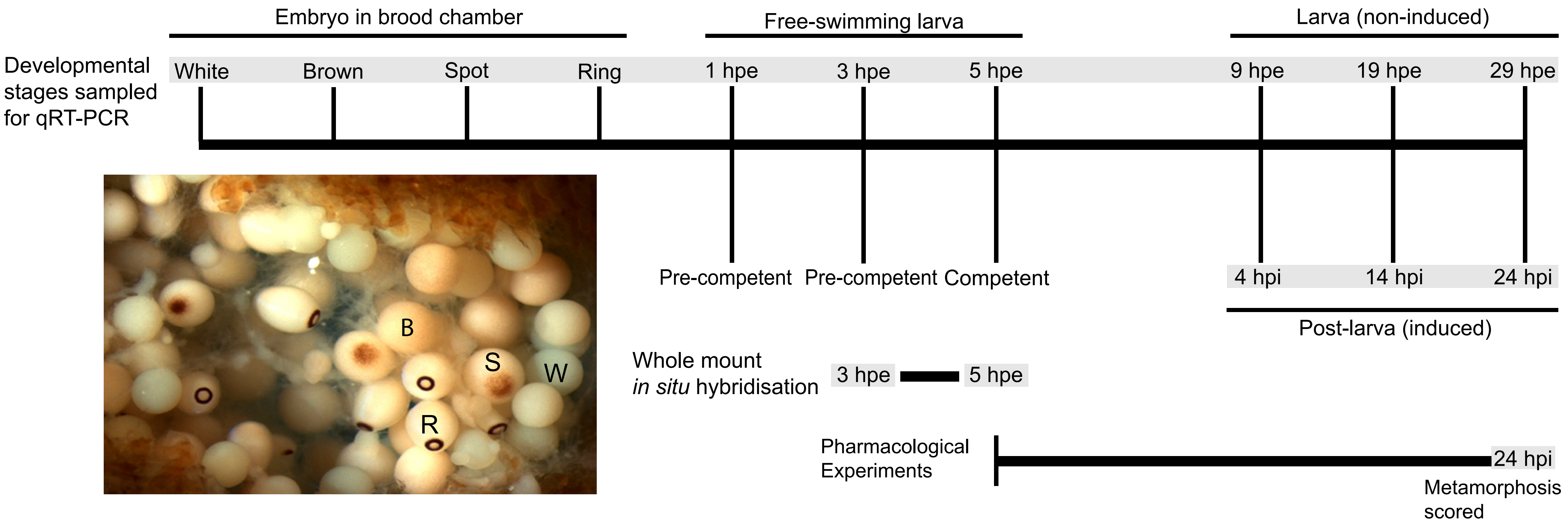

Embryo in brood chamber
Free-swimming larva
Larva (non-induced)
Developmental stages sampled for qRT-PCR
White
Brown
Spot
Ring
1 hpe
3 hpe
5 hpe
9 hpe
19 hpe
29 hpe
Pre-competent
Pre-competent
Competent
4 hpi
14 hpi
24 hpi
Post-larva (induced)
B
S
W
R
Whole mount *in situ* hybridisation
3 hpe
5 hpe
Pharmacological Experiments
24 hpi
Metamorphosis scored

Supplementary file 4. Nucleotide sequences used to design RNA probes for whole mount *in situ* hybridisation. Grey highlights indicate both the 798 bp probe and the 1649 bp probe, corresponding respectively to position 656 – 1453 and 3082 – 4730 in the open reading frame.

ATGTCTTACGATCTTATAAAAGTATCAAAGAAACCTGGTGAAAGTCTAGGGCTGGGATTCAACAAGAACTCCGAGAGACAGAACAAAGTGTATGCCATTCTTGAG
GGAACAGCACTACAGAGGTCTGGTGAAGTGAGGCCTGGTGACCTAATTGTTGAAGTTAATGGAGTTGATGTGTCTGGATATACTTCCAGTCAACTTATAGATATC
ATATCCACTCTCCAGGATAAGAGTGAAGTATCGCTAAAGATTCTCAGACCAGATGGCTCCCTGAGTAACGGTGGAACCCCAGCTGTGAGTATTGACCACCCAAAC
AACGAAACTCCTGCACCAGCTACCACTTCTCGTCCTAACCAGAGACAGAGACGTAAAGGCGGGCCTCTTCCACAGATCACAGAGACATTGGACGACGGTGGTAAA
CCGGGCGGACTACTTCAAGTGAAGGATCACTTTGCAAGTGGTAAGAGACACTCCCTCACTCCGGAGTCCTCGAGAAGGGAAATGGTACCGCTACAGCCATCAAAG
AGTTTAGATTTGGGTGCGCTGCCAACTTGGAGGAACAAGACCTTCATTACTCTTAATAACTATGTGACTGGCGAACAGCAGACGGACAGACTCCATACCCAGTCC
AAGGCAGTGCCTAAACAAACTGGATGTCATCCTTCTCATTGCGTTGGCTCAATAATTTGGCCTAAGGAGCACCCAAGGTCCAGGCCTTATGGGCCTCCAAGACCA
AAGGAAGAGATGAAAGAATTGGCTAAGGAGTACATCAAGATTTATTATGATTCTTTAAAATTTGCACTAAGTGATGATTATGAACAAAAGCTCCAATCAAGAACA
CTTCAAGTTTGCCAAGAAATAGAAGAGAAAGGTCACTATAACTTGACTTACGAGGAGCTCACCTACGGGGCACGTTTGGCATGGAGGAACGCCCCTCGCTGTGTC
AATAGGATAATATGGCGACAGCTTGAGGTACTTGATGCAAGGGAAGTAGATTCAGCTAAAGAAATGTTTGAGGCACTGTGTGATCATCTCCGCTATGCCACAAAC
CAAGGATCAATTAGGTCAACCATTACAGTATTTAGACATCGCACGAGAGCCAACTCAGACTTTAGAGTCTGGAACTCTCAGCTCATCAGATATGCCGGCTACAAG
CAACAGGATGGTTCTATAATTGGAGACCCTGACAGCGTTGAGTTCACAGAGGTTTGTCGCAAGCTGGGCTGGAGTCCCCCTAACAATAAACCCGGGATGTTTGAC
GTGTTGCCGCTGGTTCTTCAAGCAAACGGCGAACCACCTGAAATGTTCACGATCCCACCAGAACTGATAATGGAGGTCGATATTGTCCACCCAGAGTACAAGTGG
TTCAAGGATCTCAAGCTGAAGTGGTACGCTGTTCCTGGCGTCTCCAATATCCTCCTTGATATTGGAGGGCTTGAATTTACAGGTGCTCCTTTTAACGGGTGGTAC
ATGAGCACTGAGATAGCTGCGAGGAATTTCAGCGATGAATATCGTTACAATTTGCTAAAGCCTGTTGCTGAGAGGATGGGTCTGAACACATCAACCTACAAGTTA
TGGAAGGACAGGGCCCTTGTTGAACTTAACGTAGCTGTATTACATAGTTACCAGTCTGCTGGAGTTTCAATTGTTGATCACCATACTGCTACCGACGGGTTCTCA
GGGTTTTTCAAGTCAGAGAGTGCATCTCGTGGAGGGTGCCCGGCAGATTGGGTGTGGCTAGTCCCGCCCATTTCTGGCAGCGTGTCTAAACTATTCCATCAAGAA
ATGTTGCTGTATTATCTGACACCAGCTTATGAGTATCAGGAACCTGCTTATAAATATTATCACTTGCCCGGGGATATGCCAAAACTCAATACTGGAAGGACTTTC
AAGGCTCTTGCAACTATGGTCCTTGATGCCACCAGAATGATGAGGAATGTTAAAAAGAAGCGTATCAAAGCCACAGTGCTCTACGCCACTGAGACCGGCCGATCC
AAGAACTACGCCAACATAGTTAAAACCCTTTTCGACCGAACCTTCAACTGCTCCGTTTATTGCATGGACGAGTACAACAGAGCCAATCTTGAGCATGAGCAGCTG
GTTCTCATCGTTACGAGCACTTTTGGTAGTGGGGACCCACCTGCTAATGGAGAGGTAAGAGTGTATGCTGTATTTGGATTGGGGTCCCGTGCTTATCCTAACTTC
TGTGCTTTTGCTCACACAATTGACAACCTCTTCCTTTCTCTCGGGGCCGAACAGGCCTATCCTTGTGGGGAGGGAGACGAACTCTGTGGTCAAGAGGAGAGCTTC
CAGAGCTGGCTGAGAGAGTGTTACTTGAGATCTTGCGAAGTGTACAAGCTTGAGCCGAGATTAGAAGGAAGTGATTGCACTGTCGTCAAGTCCGAATACAAGAAA
GACTTTTTTAGAATTTCTTCATTTACTGAACCTGTCCCAACCAAAGACATTTGCCGAGATTTGTCTCACGTTCACAAGAAGAAGATTTATGCTGGTACTTTAGTG
TCAAGAGCTAAACTGCAGTCATCACAATCTGAGCGTAATACAATACTAGTGGTCATAAAGCCTCAGAATCACATGAATTACCAGCCTGGAGACCACATTGCCATT
TATCCTCAGAACAATCCCAGCCTGGTACGACAGCTGCTAGAGAGACTCCCCCTCACCTCCGCAATAGATGAACCAATCATCATTGAGTCCCAATATGAAGCTGAA
GGTGGCATTAAATGGAATAAGGAGAGGAGGTTACCATTTCCTGTTACTTTGCAAGAAGCCTTCATGTACTATCTTGACATTACCACTCCACCTACACCACAGCTG
CTCCAACAATTTCAAAAAATGGCCACTCGAAAGTTGGAGCAGAATTTCCTCGAGGAGCTGGGGAAAGGAGGTGACGTTTACGAGGACTGGAAGTATGAGAGGTTT
CCTAACCTTCTGGAAGTGATGGACCAATTCCATTCGCTTAAACTTGACGTGCCATTTTTACTCCAAAACCTGCCACTGCTTCAATGCCGTTACTACTCCATCAGT
TCATCCCCTAACGCTCATCCAAATGAGATTCATGCCACCATTGCCGTGGTAACCTTCAGGAAGAGAGGAGGACAGGGACCACGTCACTATGGGGTCTGTTCCACT
TGGCTCAACAAGATGGAGCCGGGATCAGAATCCATTGTACCTTTTGTGATCAGAAGGACTAATTCATTTCATATGCCAGAGGACTCTCATGCTCCTATCATAATG
GTCGGCCCTGGTACTGGGATAGCTCCTTTTAGAGGATTCTGGCAGGAAAGGATGTACCAGCGAAGCGAAGAACTCAAGAAGCAGCTTCTCTCTAAAGCAGTTCCG

GCCCGAGCTGCTCGTGTCCCTCGCAATCAAAAAGGAGGCAGGGCAGTGATCCCAGACACGCCCGCTAACAAGATAAGCATTGGAGGGGGAGGGATGGGGCAGGCT
GGTAGGAGGGGCTTTGTATCCACGGTAACGGCCCCAGTAATTAAGCTACTTGATGTTGATTCTCGACCAACTCAAGCCCCGGTCTCTGATATTGATGATACGGAG
AGCAGCAGCGAGAGTGAGAGTTCGGATGAGGAAGTAAAACGAGACAAACAAGTTCAGTTTAAAGTACCCAAAACCTCCGGCACTTTTAAACTAAGGCGCTCGGCC
TCAGATGAGTTCCAAAGGAAGGATCTTGCTAGTCTTGTGGCCGCTAGAGACAGCCACTGGGGAGACATGACCCTGTATTTCGGTTGTCGCCGTAACGACACAGAT
TACATATACAGAGAAGAGATTAAGAGAGCACAGCTGACGGGGGCAATGAATAACGTTCATGTAGCTTTCTCCAGGGAAGGGCCGCAAAAAACTTATGTCCAACAT
TTATTGAAGAAGAATGCGGACACCATAGTCCAGCAGCTGATAGAGGAGAGGGGACACTTTTATGTGTGTGGGGACGTTTCCATGGCAGCAGACGTGGGACGTACC
CTTCAGAATATATTTGAGGAGAACGCTGCAATGTCTAGTGATGAAGCGAGACAGTTAATAGAATCAATGAAAGATAATGGCTATTATCATGAAGACATCTTTGGT
GTTACTCTGAAGACTGCTGAAGTTACAAGTCGTGTCAGAAATGCAGCCAAAAAAGCATGGAGGATACTCGTTAGTGCTGCTGATCATAGCCCCATCACTCCGATG
TCTGCTCTATCCAGCAGAGCACCCATCACACCAACCACTCCTGGATTGCTTGATACCCCTCAAGACGGAACCCTTCGTTTGGGTGGAAACCGTTTCTCCAAACGC
CCGACAGTGACGGTCCTCATTCCAACGCCCACGCAGGCTAACGAGCCTACATCACCTGGCAACAGAACGTATGTTGACAAGGCAACGGAGAAAGAGGAAAGGAGG
AGGAGGAGGAGAGAGGAAAGGGAGAGGACGGAGGACAAAGAGAAAAAGGAGCGAAAGAAGATGAAGAAAACCTTGGAGAGGCTTCCGTATGAGTACCCAGTCTTG
ATGGAGCCTCCGTCTCCACCTCCAATGTCCCCGAATGTTTTTAGAAGGAGCAAAACCAGTTTTTCTAGTCCTCCCCCCTCTCCCTCTGTAGCTGGTGGCTTCGAT
TATTATTTATAAGGAGGAGACAAAACAGAATTAATAATTAGTTAATAACTTCTCATAGCTACCCCCTTAGCTGCTTATTATTACATATTATAGTTATGGTTAGCC
AAGCCTTTTTTATTTTG
